# Limb Threatening Constriction Ring Syndrome of Right Leg

**Published:** 2012-07-01

**Authors:** Farrukh Mahmood, Shehzadi Tasneem

**Affiliations:** Department of Pediatric Plastic Surgery, The Children’s Hospital and the Institute of Child Health Lahore, Pakistan

**Figure:**
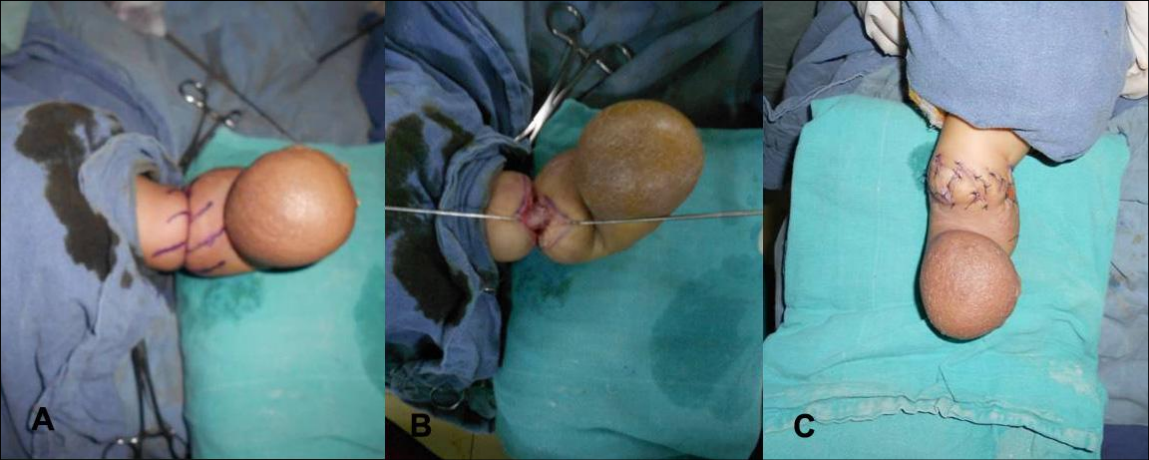
Figure 1: Various steps of multiple Z-plasties. (A) Marking, (B) Release (C) Z-plasties.

**Figure:**
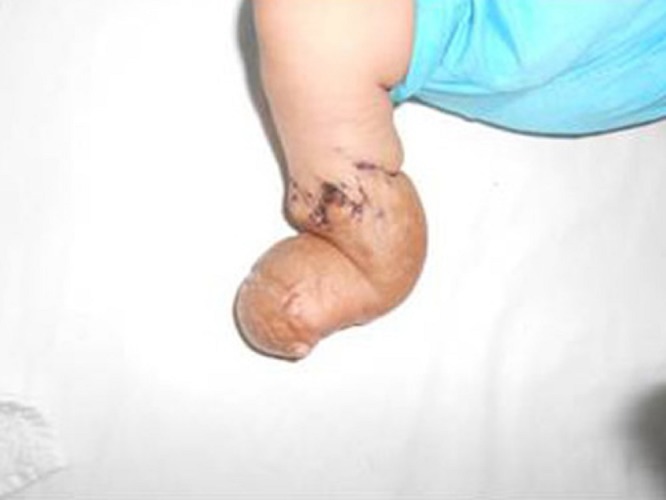
Figure 2: At two weeks follow up- lymphovenous edema had reduced by 40%.

A 25-day-old male neonate was received in our outpatient department with constriction ring of right leg with bluish discoloration and swelling of foot and acro-syndactly of both hands. The constriction ring was threatening to the limb that already had lost digits as an in-utero event. Multiple Z-Plasties of half circumference of leg was done with release of constriction band. Wound was stitched with 5/0 vicryl rapide (Fig.1). Patient had uneventful recovery and swelling quickly subsided by 40% during two weeks of the operation (Fig.2). The patient is under close surveillance for need of multiple Z-plasties of the remaining half of the constriction ring.

Constriction ring syndrome (CRS) is a rare congenital anomaly characterized by partial or complete constriction anywhere around the limb. It can lead to limb threatening complications. CRS was first described by Van Helmont in 1689 as intrauterine amputation and Montgomery in 1832 as free strands of tissue. Estimated incidence is approximately 1 in 10,000 live births with equal sex distribution. Malformation can be classified into five groups which aids in determining the timing and type of surgical management. Club foot, facial clefts, cleft lip and palate, body wall defects, and cutis aplasia are common associations. Tight constriction ring leads to distal lymphovenous obstruction and threat to the distal extremity [1,2]. Our case of CRS with distal obstruction was presented with limb threatening situation where extremity was salvaged by immediate surgical intervention.

## Footnotes

**Source of Support:** Nil

**Conflict of Interest:** Nil

